# Trends and predictive factors for treatment failure following artemisinin-based combination therapy among children with uncomplicated malaria in Ghana: 2005–2018

**DOI:** 10.1186/s12879-021-06961-4

**Published:** 2021-12-15

**Authors:** Benjamin Abuaku, Nancy Odurowah Duah-Quashie, Neils Quashie, Akosua Gyasi, Patricia Opoku Afriyie, Felicia Owusu-Antwi, Anita Ghansah, Keziah Laurencia Malm, Constance Bart-Plange, Kwadwo Ansah Koram

**Affiliations:** 1 Department of Epidemiology, Noguchi Memorial Institute for Medical Research, University of Ghana, Legon, Accra, Ghana; 2 Centre for Tropical Clinical Pharmacology and Therapeutics, University of Ghana Medical School, Accra, Ghana; 3 National Malaria Control Programme, Public Health Division, Ghana Health Service, Accra, Ghana; 4 World Health Organization, Country Office, Accra, Ghana; 5 Department of Parasitology, Noguchi Memorial Institute for Medical Research, University of Ghana, Legon, Accra, Ghana

**Keywords:** Trends, Predictive factors, ACTs, Treatment failure, Ghana

## Abstract

**Background:**

Since the introduction of artemisinin-based combination therapy (ACT) in Ghana in 2005 there has been a surveillance system by the National Malaria Control Programme (NMCP) and the University of Ghana Noguchi Memorial Institute for Medical Research (UG-NMIMR) to monitor the therapeutic efficacy of ACTs for the treatment of uncomplicated malaria in the country. We report trends and determinants of failure following treatment of Ghanaian children with artesunate-amodiaquine (ASAQ) and artemether-lumefantrine (AL) combinations.

**Methods:**

Per protocol analyses as well as cumulative incidence of day 28 treatment failure from Kaplan Meier survival analyses were used to describe trends of failure over the surveillance period of 2005–2018. Univariable and multivariable cox regression analyses were used to assess the determinants of treatment failure over the period.

**Results:**

Day 28 PCR-corrected failure, following treatment with ASAQ, significantly increased from 0.0% in 2005 to 2.0% (95% CI: 1.1–3.6) in 2015 (p = 0.013) but significantly decreased to 0.4% (95% CI: 0.1–1.6) in 2018 (p = 0.039). Failure, following treatment with AL, decreased from 4.5% (95% CI: 2.0–9.4) in 2010 to 2.7% (95% CI: 1.4–5.1) in 2018, though not statistically significant (p = 0.426). Risk of treatment failure, from multivariable cox regression analyses, was significantly lower among children receiving ASAQ compared with those receiving AL (HR = 0.24; 95% CI: 0.11–0.53; p < 0.001); lower among children with no parasitaemia on day 3 compared with those with parasitaemia on day 3 (HR = 0.02; 95% CI: 0.01–0.13; p < 0.001); and higher among children who received ASAQ and had axillary temperature ≥ 37.5 °C on day 1 compared with those with axillary temperature < 37.5 °C (HR = 3.96; 95% CI: 1.61–9.75; p = 0.003).

**Conclusions:**

Treatment failures for both ASAQ and AL have remained less than 5% (below WHO’s threshold of 10%) in Ghana since 2005. Predictors of treatment failure that need to be considered in the management of uncomplicated malaria in the country should include type of ACT, day 3 parasitaemia, and day 1 axillary temperature of patients being treated.

**Supplementary Information:**

The online version contains supplementary material available at 10.1186/s12879-021-06961-4.

## Background

Since the discovery of malaria parasites by Charles Louis Alphonse Laveran in 1880 [1], malaria has remained one of the major public health problems in the world, particularly, in sub-Saharan Africa (SSA). The first comprehensive malaria report in 2005 by the World Health Organization (WHO) and the United Nations Children’s Fund (UNICEF), under the Roll Back Malaria (RBM) initiative, estimated that 300–500 million clinical malaria episodes occurred annually in the world [2]. About 60% of the estimated global malaria cases as well as 80% of all malaria deaths were reported to have occurred in SSA [2]. In 2018, global malaria cases were estimated to be 206–258 million with 93% of the cases and 94% of deaths occurring in SSA [3]. In 2019, Ghana was classified as one of the 11 malaria high burden to high impact (HBHI) countries in the world with 500,000 more cases in 2018 compared with 2017 [3]. This notwithstanding, malaria parasite prevalence among children under 5 years old in Ghana declined from 27% in 2014 to 14% in 2019 [4].

Prompt and effective treatment of uncomplicated malaria remains one of the key interventions within the Global Technical Strategy for Malaria (2016–2030) because it has the advantage of preventing the progression to severe illness and death [5]. Since April 2001 the WHO has recommended the use of Artemisinin-based combination therapies (ACTs) for treating uncomplicated *Plasmodium falciparum* malaria [6, 7]. The ACTs have the advantage of preventing the development and spread of resistance by reducing parasite biomass, gametocyte carriage and transmissibility; rapid elimination resulting in minimal selective pressure; and rapid clinical relief [8, 9].

Ghana adopted the use of ACTs in 2004 when it had become clear that the therapeutic efficacies of chloroquine and sulphadoxine/pyrimethamine, which were the first- and second-line drugs for uncomplicated malaria, were less than 70% [10]. In 2005 artesunate-amodiaquine (ASAQ) combination was rolled out as treatment for uncomplicated malaria in the country. In 2008, artemether-lumefantrine (AL) combination and dihydroartemisinin-piperaquine (DHAP) were adopted as alternate first-line antimalarials for persons unable to tolerate ASAQ [11]. Currently, ASAQ and AL remain alternate first-line antimalarials for uncomplicated malaria in Ghana with DHAP being the second-line antimalarial [12].

**Fig. 1:**
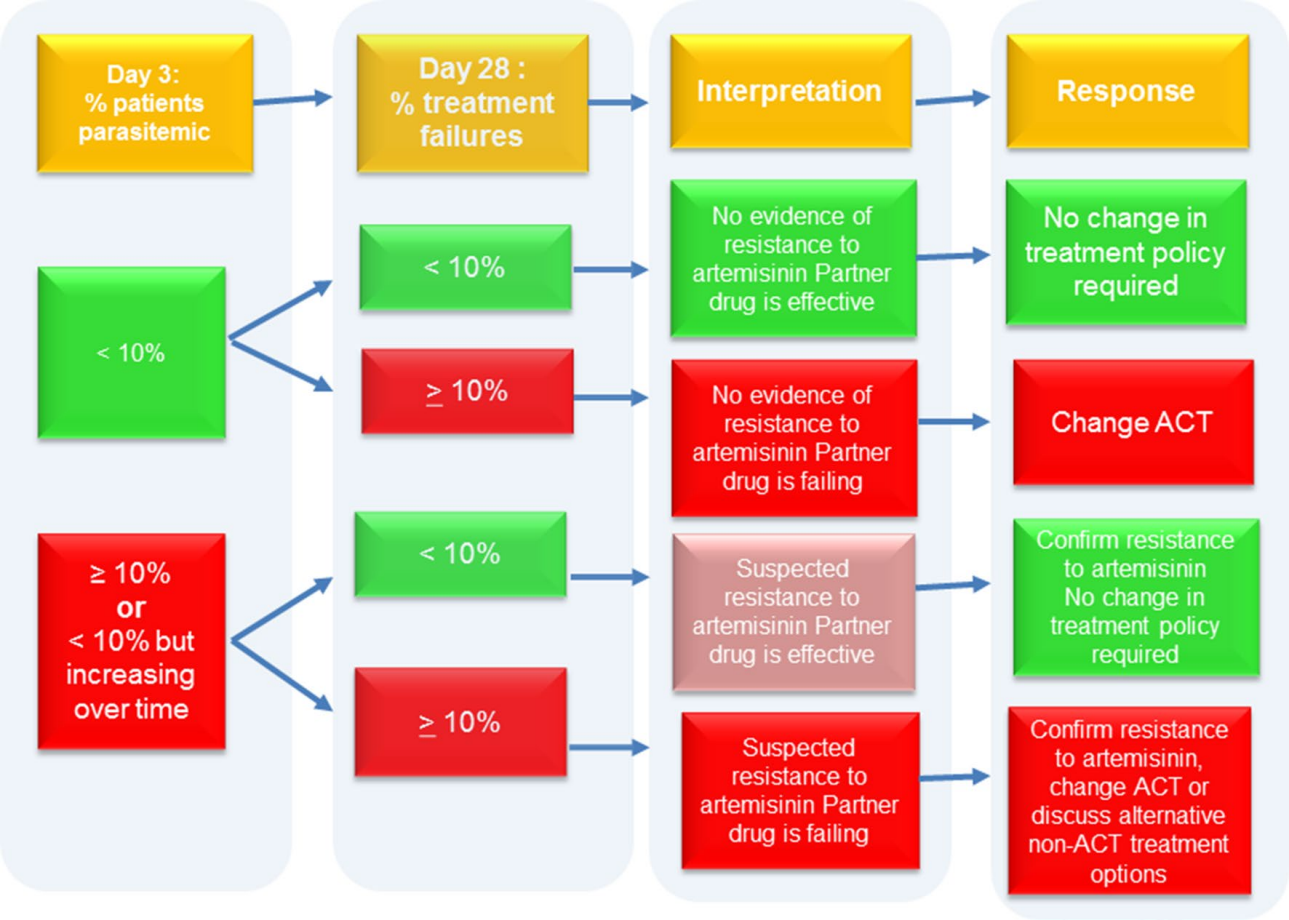
Decision-making process on TES results [22]

Following the introduction of ACTs in 2005, a surveillance system was established by the National Malaria Control Programme (NMCP), in collaboration with the University of Ghana Noguchi Memorial Institute for Medical Research (UG-NMIMR), to continuously monitor the therapeutic efficacies of ACTs to inform antimalaria drug policy in the country using the recommended WHO criteria, which has the proportion of patients parasitaemic on day 3 as indicator of suspected artemisinin partial resistance and the proportion of treatment failure by day 28 or 42 (depending on half-life of ACT partner drug) as indicator of partner drug resistance (Fig. 1). We report trends and determinants of treatment failure following ASAQ and AL treatment of children with uncomplicated malaria in ten sentinel sites across Ghana using surveillance data gathered between 2005 and 2018 (Table 1).

**Table 1:** Details of data analyzed

Year of study	Test medicines	Number Enrolled	Age range	Sponsor	Status of data	Data source
2005	ASAQ: *Non-fixed combination*	543	6 mths–59 mths	^*^GF	Published [36, 37]	9 sites
2007	ASAQ: *Non-fixed combination*	496	6 mths–59 mths	^*^GF	Published [37]	9 sites
2010	ASAQ: *Fixed combination*	126	6 mths–9 yrs	^*^GF	Published [37]	3 sites
	AL: *Fixed combination*	181	6 mths–9 yrs	^*^GF	Published [37, 37]	5 sites
2012	ASAQ: *Fixed combination*	164	6 mths–9 yrs	^+^NAMRU-3	Unpublished	3 sites
2013	ASAQ: *Fixed combination*	159	6 mths–9 yrs	^*^GF	Published [39]	4 sites
	AL: *Fixed combination*	171	6 mths–9 yrs	^*^GF	Published [39]	3 sites
2014	ASAQ: *Non-fixed combination*	191	6 mths–14 yrs	^+^NIH	Published [16]	2 sites
2015	ASAQ: *Fixed combination*	492	6 mths–9 yrs	^*^GF	Published [13]	9 sites
	ASAQ: *Non-fixed combination*	237	6 mths–14 yrs	^+^NIH	Unpublished	2 sites
	AL: *Fixed combination*	472	6 mths–9 yrs	^*^GF	Published [13]	8 sites
2016	ASAQ: *Non-fixed combination*	141	6 mths–14 yrs	^+^NIH	Unpublished	2 sites
2017	ASAQ: *Non-fixed combination*	192	6 mths–14 yrs	^+^NIH	Unpublished	2 sites
2018	ASAQ: *Fixed combination*	692	6 mths–9 yrs	^*^GF	Unpublished	9 sites
	AL: *Fixed combination*	653	6 mths–9 yrs	^*^GF	Unpublished	10 sites

*ASAQ* Artesunate-Amodiaquine combination, *AL* Artemether-Lumefantrine combination, *GF* Global Fund, *NAMRU-3:* Naval Medical Research unit 3, *NIH* National Institute of Health; *Routine surveillance years with 2–3 years interval; ^+^Part of in-vitro/ex-vivo studies

## Methods

### Study sites

**Fig. 2:**
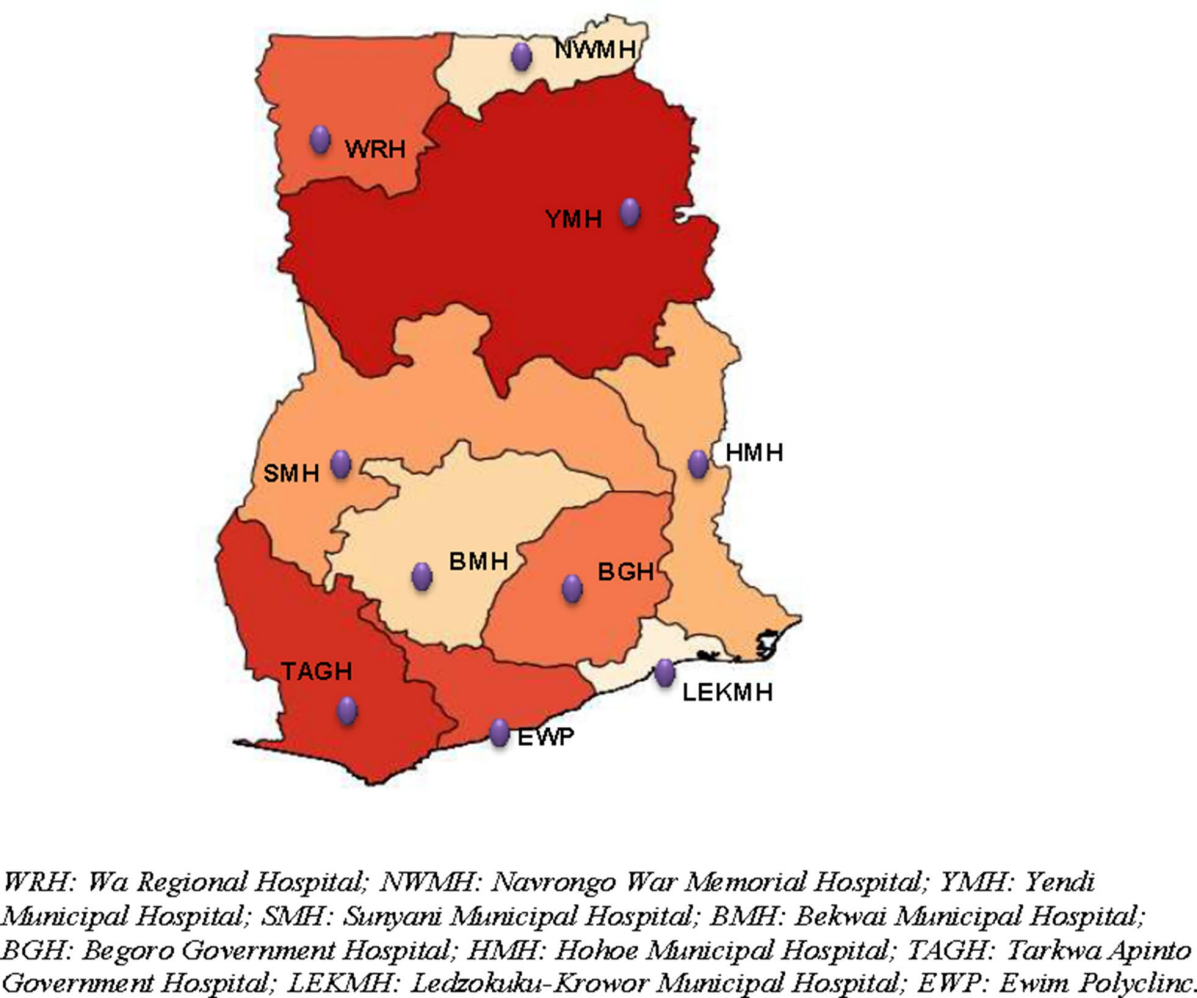
Map of Ghana showing TES sentinel sites in the previous ten regions

Since 2005, ACT efficacy studies have been conducted in purposively selected ten (10) sentinel sites across Ghana. These sites represent the previously ten administrative regions of the country and the three main ecological zones: savannah, forest, and coastal. Three of these sites (Navrongo War Memorial Hospital, Yendi Municipal Hospital, and Wa Regional Hospital) are located within the northern belt, which is savannah; four (Sunyani Municipal Hospital, Bekwai Municipal Hospital, Begoro Government Hospital, and Hohoe Municipal Hospital) are located within the middle belt, which is forest; and three are located within the southern belt, which is forest (one site: Tarkwa Apinto Government Hospital) and coastal (two sites: Ledzokuku Krowor Municipal Hospital and Ewim Polyclinic) (Fig. 2). The sites have been described elsewhere [13].

### Study design

Studies over the period have been one-arm, prospective, evaluation of the clinical, parasitological, and haematological responses to ASAQ or AL administered to children by the directly observed therapy (DOT) approach using WHO 2003 and 2009 protocols [14, 15].

### Inclusion criteria

A summary of specific inclusion criteria adopted for surveillance activities from 2005 to 2009 using the WHO 2003 protocol include: children aged 6 months to 59 months; axillary temperature ≥ 37.5 °C; and asexual parasitaemia of 2,000–200,000/µL. A summary of specific inclusion criteria adopted for surveillance activities from 2010 to 2018 using the WHO 2009 protocol include: children aged 6 months to 9 years (extended to 14 years in some studies) (Table 1); axillary temperature ≥ 37.5 °C or history of fever during the past 24 h; asexual parasitaemia of 1,000–250,000/µL. A summary of common inclusion criteria adopted for the two periods include: mono-infection with *P. falciparum* detected by microscopy; ability to swallow oral medication; parental consent; parent/guardian willing to comply with study protocol for the duration of the study; and parent/guardian willing to comply with follow-up schedule.

### Exclusion criteria

A summary of exclusion criteria for the two periods include: general danger signs or signs of severe falciparum malaria; mixed or mono-infection with other Plasmodium species detected by microscopy; severe malnutrition; other non-malaria febrile conditions; known underlying chronic or severe diseases; regular medication likely to interfere with antimalarial activities; and history of hypersensitivity to test medicines.

### Treatment, follow-up, and laboratory procedures

All treatment given followed the DOT approach. There were fixed and non-fixed combination therapies (Table 1). The follow-up schedule and laboratory procedures have been published elsewhere. Prior to 2014 merozoite surface proteins 1 and 2 (*msp1, msp2*) and glutamate-rich protein (*glurp*) were used to distinguish between re-infection and recrudescence as recommended by WHO [15–19]. From 2014 to 2018 only *msp2* was used based on the observation that *msp1* was less discriminatory and that *glurp* was prone to “artefact bands” [13, 20, 21].

### Data analysis

The main outcome variable was treatment failure from data collected during routine surveillance years (2–3 years interval) and years when in-vitro/ex-vivo studies were being conducted in some sentinel sites (Table 1). As per WHO classifications, treatment failure is a combination of early treatment failure (ETF), late parasitological failure (LPF) and late clinical failure (LCF) [15]. ETF is defined as danger signs or severe malaria on days 1, 2 or 3, in the presence of parasitaemia; parasitaemia on day 2 higher than on day 0, irrespective of axillary temperature; parasitaemia on day 3 with axillary temperature ≥ 37.5 °C; and parasitaemia on day 3 ≥ 25% of count on day 0 [15]. LTF is defined as danger signs or severe malaria in the presence of parasitaemia on any day between day 4 and day 28 in patients who did not previously meet any of the criteria of early treatment failure; and presence of parasitaemia on any day between day 4 and day 28 with axillary temperature ≥ 37.5 °C in patients who did not previously meet any of the criteria of early treatment failure [15]. LPF is defined as presence of parasitaemia on any day between day 7 and day 28 with axillary temperature < 37.5 °C in patients who did not previously meet any of the criteria of early treatment failure or late clinical failure [15].

Per protocol analyses as well as cumulative incidence of treatment failure from Kaplan Meier survival analyses were used to describe trends of PCR-uncorrected and PCR-corrected day 28 treatment failure over the surveillance period. The estimates of treatment failure were obtained from the WHO Excel® data template used in the studies [15]. Data for ASAQ trend analysis were from seven (7) sites (Navrongo War Memorial Hospital, Wa Regional Hospital, Sunyani Municipal Hospital, Bekwai Municipal Hospital, Begoro Government Hospital, Tarkwa Apinto Government Hospital, and Ewim Polyclinic) that consistently generated data for four (4) surveillance years (2005, 2007, 2015, and 2018) whilst data for AL trend analysis were from five (5) sites (Navrongo War Memorial Hospital, Sunyani Municipal Hospital, Bekwai Municipal Hospital, Begoro Government Hospital, and Ewim Polyclinic) that consistently generated data for three (3) surveillance years (2010, 2015, and 2018).

Univariable analysis was performed using the Cox Proportional Hazard Model (CPHM) to assess the association between each covariate and treatment failure and to determine variables to be considered in a multivariable analysis based on a significance level of less than or equal to 20%. Covariates considered for treatment failure, using pooled data from all ten (10) study sites, were gender, age, ecological zone, drug type, parasite density at enrolment (day 0), parasitaemia on day 3, vomiting at least once during the three days of treatment, axillary temperature on day 0, and axillary temperature on day 1. Variables that were not statistically significant but considered to be clinically important were included in the adjusted or multivariable model.

Multivariable cox regression analysis was used to determine the simultaneous effect of multiple risk factors on treatment failure. Analyses were done for the overall pooled ASAQ and AL data as well as separately pooled data for ASAQ and AL. The Hazard ratio, which is the exponent of each regression coefficient was used in the interpretation of model results. All tests were conducted with 95% confidence interval. P-values ≤ 0.05 were considered statistically significant. A cluster term was included in the model to account for clustering of data by study site. All cox regression analyses were carried out using R software (Version 4.0.2). Proportional hazard assumption test performed showed no violations at 5% significance level (Additional file 1).

## Results

### Demographic, clinical, and parasitological characteristics of study participants

**Fig. 3:**
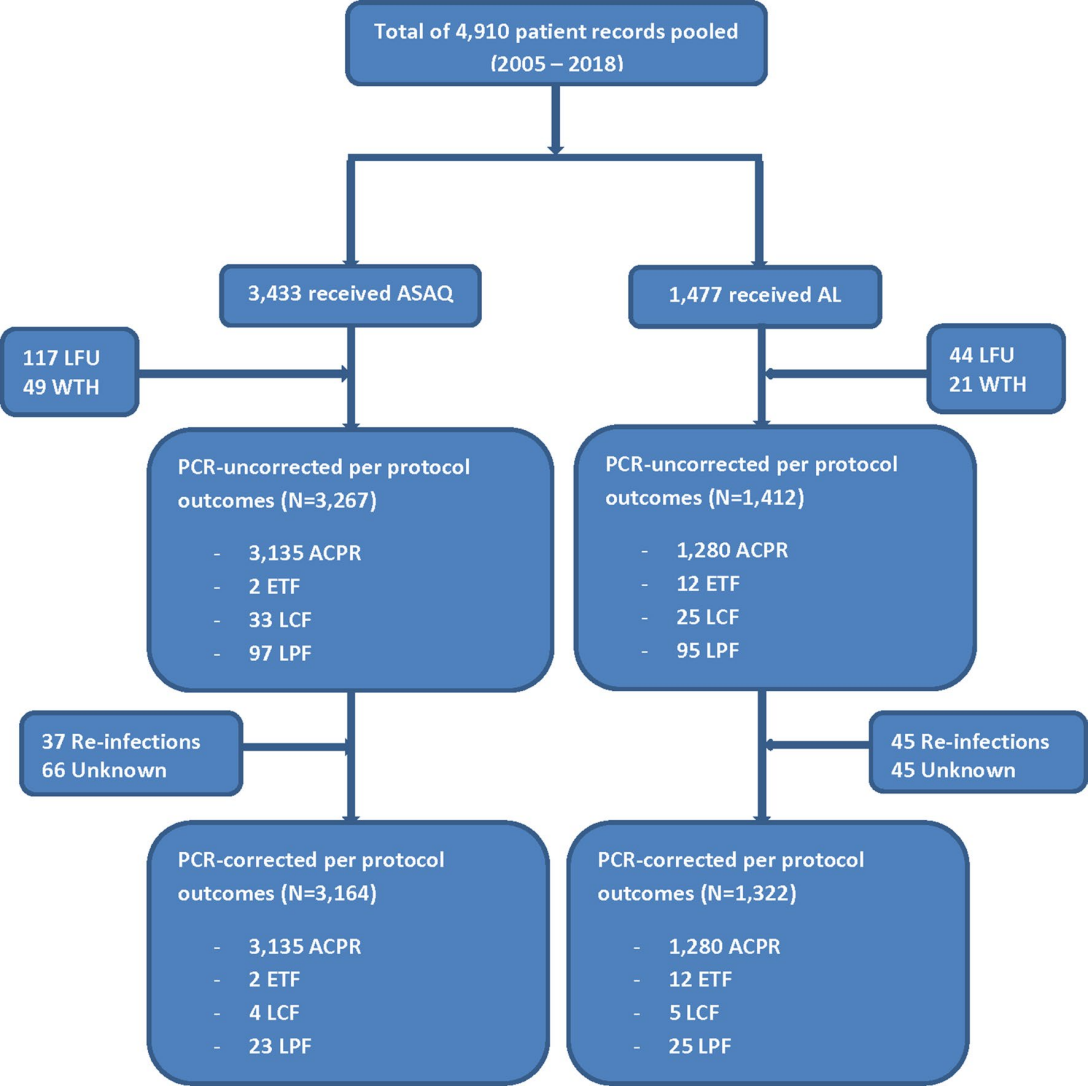
Participant flow showing number analyzed and per protocol treatment outcomes. *ASAQ* Artesunate-amodiaquine, *AL* Artemether-lumefantrine; *LFU* Loss to follow-up, *WTH* Withdrawn, *ACPR* Adequate clinical and parasitological response, *ETF* Early treatment failure; *LCF* Late clinical failure, *LPF* Late parasitological failure

A total of 4,910 participants were pooled from the different studies conducted between 2005 and 2018. The majority of participants were male (52.8%), < 5 years old (60.7%), exposed to ASAQ treatment (69.9%), had axillary temperature ≥ 37.5 °C on day of enrolment (79.7%), had axillary temperature < 37.5% °C on day 1 post-treatment (91.5%), had axillary temperature < 37.5% °C on day 2 post-treatment (91.5%), had axillary temperature < 37.5% ^°^C on day 3 post-treatment (99.2%), did not vomit during treatment (86.7%), had parasite density < 50,000/µL (55.1%), had no parasitaemia on day 2 post-treatment (97.0%), and no parasitaemia on day 3 post-treatment (99.7%) (Table 2 and Fig. 3). Proportion of participants with parasitaemia on day 3 increased from 0.2% (95% CI: 0.01–1.24) in 2005 to 0.6% (95% CI: 0.28–1.23) in 2018, but the increase was not statistically significant (p = 0.455).

**Table 2:** Demographic, clinical, and parasitological characteristics of study participants N = 4910

Characteristics	n	%
Gender		
Male	2595	52.9
Female	2315	47.1
Age group (yrs)		
< 5	2982	60.7
≥ 5	1928	39.3
Ecological zone		
Savannah	1475	30.0
Forest	2396	48.8
Coastal	1039	21.2
Antimalarial drug administered		
AL	1477	30.1
ASAQ	3433	69.9
Temperature (Day 0)		
< 37.5 °C	999	20.3
≥ 37.5 °C	3911	79.7
Temperature (Day 1)		
< 37.5 °C	4494	91.5
≥ 37.5 °C	416	8.5
Temperature (Day 2)		
< 37.5 °C	4859	99.0
≥ 37.5 °C	51	1.0
Temperature (Day 3)		
< 37.5 °C	4872	99.2
≥ 37.5 °C	38	0.8
Vomited at least once during treatment		
No	4258	86.7
Yes	652	13.3
Parasite density/µL (Day 0)		
< 50,000	2703	55.1
≥ 50,000	2207	44.9
Absence of parasitaemia (Day 2)		
No	146	3.0
Yes	4764	97.0
Absence of parasitaemia (Day 3)		
No	15	0.3
Yes	4895	99.7

### Trends of treatment failure

**Fig. 4:**
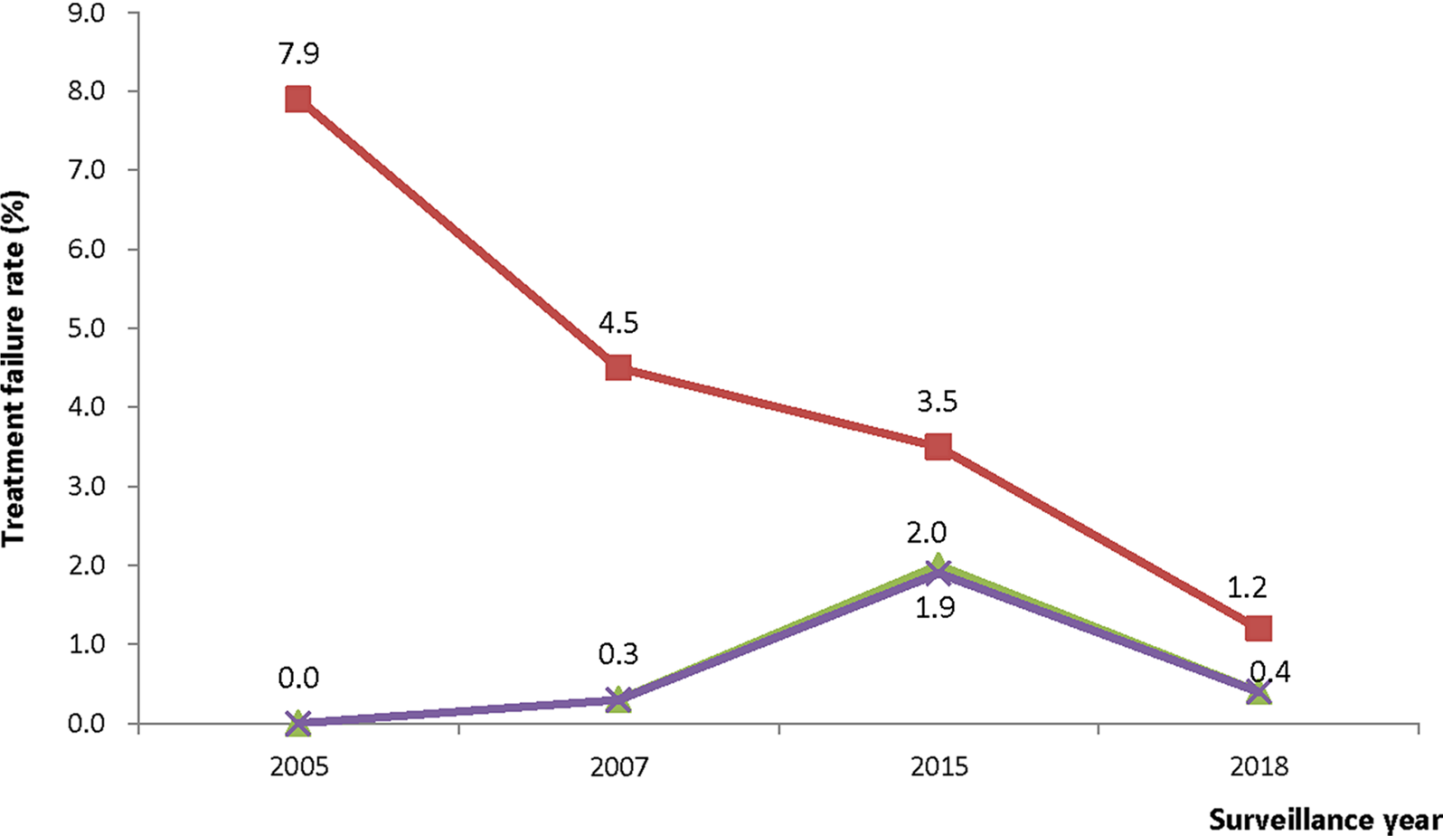
Trends of ASAQ treatment failure in Ghana (2005–2018). Blue line represents PCR-uncorrected per protocol failure following treatment with ASAQ; Red represents PCR-uncorrected Kaplan–Meier survival failure following treatment with ASAQ; Green represents PCR-corrected per protocol failure following treatment with ASAQ; Purple represents PCR-corrected Kaplan–Meier survival failure following treatment with ASAQ

Following treatment with ASAQ, PCR-uncorrected per protocol failure on day 28 (post-treatment) significantly declined from 7.9% (95% CI: 5.6–11.0) in 2005 to 3.5% (95% CI: 2.3–5.4) in 2015 (p = 0.003), and significantly declined further to 1.2% (95% CI: 0.5–2.8) in 2018 (p = 0.025). PCR-corrected treatment failure on day 28 (post-treatment) significantly increased from 0.0% in 2005 to 2.0% (95% CI: 1.1 -3.6) in 2015 (p = 0.013), but significantly decreased to 0.4% (95% CI: 0.1–1.6) in 2018 (p = 0.039). Similar patterns were observed with KM analysis (Fig. 4).

**Fig. 5:**
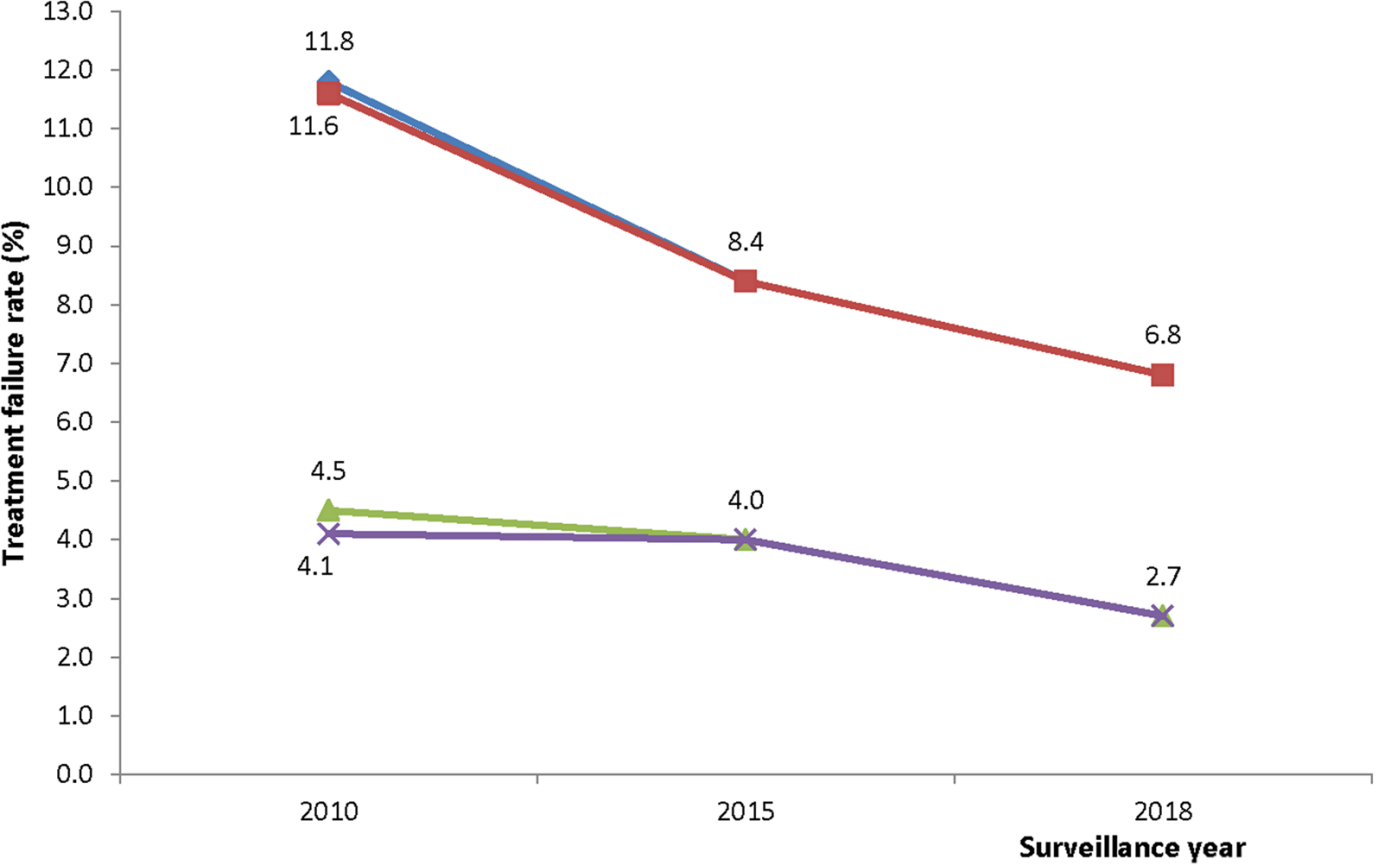
Trends of AL treatment failure in Ghana (2010–2018). Blue line represents PCR-uncorrected per protocol failure following treatment with AL; Red represents PCR-uncorrected Kaplan–Meier survival failure following treatment with AL; Green represents PCR-corrected per protocol failure following treatment with AL; Purple represents PCR-corrected Kaplan–Meier survival failure following treatment with AL

Following treatment with AL, PCR-uncorrected per protocol failure on day 28 (post-treatment) declined from 11.8% (95% CI: 7.5–17.9) in 2010 to 6.8% (95% CI: 4.6–9.9) in 2018 but this was not statistically significant (p = 0.073). Similarly, PCR-corrected treatment failure declined from 4.5% (95% CI: 2.0 -9.4) in 2010 to 2.7% (95% CI: 1.4–5.1) in 2018 but this was not statistically significant (p = 0.426). Similar patterns were observed with KM analysis (Fig. 5).

### Univariable analysis of factors associated with treatment failure

Univariate cox regression analyses of the overall pooled data showed that PCR-uncorrected treatment failure was significantly higher among patients less than 5 years old compared with those aged 5 years and above (HR = 1.55; 95% CI: 1.15–2.09; p = 0.004); significantly lower among patients without no parasitaemia on day 3 compared with those with parasitaema (HR = 0.08; 95% CI: 0.02–0.30); significantly lower among patients who received ASAQ treatment compared with those who received AL treatment (HR = 0.42; 95% CI: 0.24–0.74; p = 0.003); and significantly lower among patients who vomited at least once during the three days of treatment compared with those who did not vomit (HR = 0.60; 95% CI: 0.39–0.92; p = 0.018). PCR-corrected treatment failure was significantly higher in the coastal zone compared with the savannah zone (HR = 1.47; 95% CI: 1.09–1.99; p = 0.012); significantly lower among patients with no parasitaemia on day 3 compared with those with parasitaemia (HR = 0.03; 95% CI: 0.01–0.12; p < 0.001); and significantly lower among patients who received ASAQ treatment compared with those who received AL treatment (HR = 0.28; 95% CI: 0.14–0.56; p < 0.001) (Table 3).

**Table 3:** Overall univariable and multivariable cox proportional hazards model for PCR uncorrected/corrected treatment failure (ASAQ and AL)

Characteristics	PCR uncorrected analysis				PCR corrected analysis			
	Unadjusted		Adjusted		Unadjusted		Adjusted	
	HR (95% CI)	P-value	HR (95% CI)	P-value	HR (95% CI)	P-value	HR (95% CI)	P-value
Gender								
Female^a^								
Male	0.99 [0.84, 1.16]	0.877	1.03 [0.86, 1.24]	0.753	1.15 [0.73, 1.81]	0.556	1.31 [0.81, 2.09]	0.264
Age group (yrs)								
≥ 5^a^								
< 5	1.55 [1.15, 2.09]	0.004	1.55 [1.20, 2.02]	< 0.001	1.09 [0.74, 1.59]	0.667	1.09 [0.76, 1.55]	0.635
Ecological zone		0.007		< 0.001		0.020		0.002
Savannah^a^								
Forest Zone	0.70 [0.46, 1.08]	0.109	0.71 [0.51, 1.00]	0.052	0.68 [0.32, 1.43]	0.306	0.66 [0.38, 1.16]	0.148
Coastal Zone	1.08 [0.87, 1.34]	0.480	1.27 [1.04, 1.55]	0.021	1.47 [1.09, 1.99]	0.012	1.89 [1.52, 2.37]	< 0.001
Temperature day 0								
< 37.5 °C^a^								
≥ 37.5 °C	1.21 [0.75, 1.95]	0.426	1.27 [0.88, 1.83]	0.210	0.89 [0.52, 1.52]	0.659	0.97 [0.54, 1.75]	0.917
Temperature day 1			-					
< 37.5 °C^a^								
≥ 37.5 °C	0.95 [0.68, 1.35]	0.792	0.66 [0.49, 0.90]	0.009	1.39 [0.78,2.46]	0.264	0.85 [0.43, 1.70]	0.652
Parasitemia day 0								
< 50000^a^								
≥ 50,000	0.92 [0.67,1.27]	0.604	0.87 [0.61, 1.25]	0.457	0.88 [0.50, 1.56]	0.669	0.80 [0.44, 1.46]	0.472
Parasitaemia day 3								
Yes^a^								
No	0.08 [0.02, 0.30]	< 0.001	0.08 [0.02, 0.35]	< 0.001	0.03 [0.01, 0.12]	< 0.001	0.02 [0.01, 0.13]	< 0.001
Drug type						-		
AL^a^								
AS + AQ	0.42 [0.24, 0.74]	0.003	0.36 [0.21, 0.62]	< 0.001	0.28 [0.14, 0.56]	< 0.001	0.24 [0.11, 0.53]	< 0.001
Vomit at least once								
No^a^								
Yes	0.60 [0.39, 0.92]	0.018	0.63 [0.39, 1.02]	0.060	0.72 [0.27, 1.93]	0.514	0.81 [0.29, 2.32]	0.698

^a^Reference category

Univariate cox regression analyses of the pooled ASAQ data showed that PCR-uncorrected treatment failure was significantly higher among patients less than 5 years compared with those aged 5 years and above (HR = 1.91; 95% CI: 1.40–2.61; p < 0.001); significantly higher among patients with axillary temperature on day 0 ≥ 37.5 °C compared with those with temperature < 37.5 °C (HR = 1.73; 95% CI: 1.12–2.68; p = 0.013); and significantly lower among patients with no parasitaemia on day 3 compared with those with parasitaemia (HR = 0.13; 95% CI: 0.03–0.56; p = 0.006). PCR-corrected ASAQ treatment failure was significantly higher among patients with axillary temperature on day 1 ≥ 37.5 °C compared with those with temperature < 37.5 °C (HR = 3.36; 95% CI: 1.32–8.55; p = 0.011); and significantly lower among patients with no parasitaemia on day 3 compared with those with parasitaemia (HR = 0.05; 95% CI: 0.01–0.57; p = 0.015) (Table 4).

**Table 4:** Univariable and multivariable cox proportional hazards model for PCR-uncorrected/corrected treatment failure (ASAQ)

Characteristics	PCR uncorrected analysis				PCR corrected analysis			
	Unadjusted		Adjusted		Unadjusted		Adjusted	
	HR (95% CI)	P-value	HR (95% CI)	P-value	HR (95% CI)	P-value	HR (95% CI)	P-value
Gender								
Female^a^								
Male	1.06 [0.87, 1.28]	0.593	1.09 [0.90, 1.32]	0.355	1.09 [0.71, 1.67]	0.707	1.20 [0.90, 1.61]	0.216
Age group (yrs)								
≥ 5^a^								
< 5	1.91 [1.40, 2.61]	< 0.001	1.84 [1.48, 2.28]	< 0.001	1.41 [0.99, 2.05]	0.056	1.39 [0.83, 2.33]	0.205
Ecological zone		0.014		0.014		0.004		0.004
Savannah^a^								
Forest	0.65 [0.30, 1.39]	0.270	0.74 [0.36, 1.53]	0.419	0.30 [0.06, 1.38]	0.122	0.31 [0.07, 1.36]	0.119
Coastal	1.19 [0.59, 2.41]	0.625	1.34 [0.68, 2.65]	0.401	1.41 [0.65, 3.05]	0.383	1.70 [0.82, 3.52]	0.154
Temperature day 0								
< 37.5 °C^a^								
≥ 37.5 °C	1.73 [1.12, 2.68]	0.013	1.45 [0.89, 2.39]	0.139	0.86 [0.46, 1.62]	0.644	0.69 [0.30, 1.54]	0.361
Temperature day1								
< 37.5 °C^a^								
≥ 37.5 °C	1.06 [0.50, 2.25]	0.887	1.13 [0.53, 2.43]	0.746	3.36 [1.32, 8.55]	0.011	3.96 [1.61, 9.75]	0.003
Parasitemia day 0								
< 50000^a^								
≥ 50,000	0.80 [0.56, 1.15]	0.225	0.78 [0.54, 1.12]	0.177	0.63 [0.35, 1.12]	0.112	0.63 [0.36, 1.10]	0.106
Parasitaemia day 3								
Yes^a^								
No	0.13 [0.03, 0.56]	0.006	0.15 [0.03, 0.72]	0.017	0.05 [0.01, 0.57]	0.015	0.05 [0.01, 0.48]	0.009
Vomit at least once								
No^a^								
Yes	0.56 [0.30, 1.04]	0.065	0.59 [0.32, 1.11]	0.101	1.08 [0.23, 5.09]	0.925	1.06 [0.25, 4.42]	0.938

^a^Reference category

Univariate cox regression analyses of the pooled AL data showed that PCR-uncorrected and PCR-corrected treatment failure were significantly lower among patients with no parasitaemia on day 3 compared with those with parasitaemia (HR = 0.05; 95% CI: 0.01–0.40; p = 0.004) and (HR = 0.02; 95% CI: 0.01–0.10; p < 0.001), respectively (Table 5).

**Table 5:** Univariable and multivariable cox proportional hazards model for PCR-uncorrected/corrected treatment failure (AL)

Characteristics	PCR uncorrected analysis				PCR corrected analysis			
	Unadjusted		Adjusted		Unadjusted		Adjusted	
	HR (95% CI)	P-value	HR (95% CI)	P-value	HR (95% CI)	P-value	HR (95% CI)	P-value
Gender								
Female^a^								
Male	0.94 [0.72,1.23]	0.649	0.98 [0.71, 1.34]	0.887	1.21 [0.56, 2.61]	0.633	1.27 [0.65, 2.47]	0.477
Age group (yrs)								
≥ 5^a^								
< 5	1.36 [0.86, 2.14]	0.183	1.35 [0.89, 2.05]	0.156	0.98 [0.47, 2.03]	0.951	1.03 [0.59, 1.80]	0.913
Ecological zone		0.017		0.016		0.107		0.109
Savannah zone^a^								
Forest zone	0.66 [0.25, 1.74]	0.405	0.71 [0.27, 1.90]	0.499	0.89 [0.29, 2.73]	0.839	0.94 [0.33, 2.68]	0.911
Coastal zone	1.22 [0.56, 2.66]	0.619	1.26 [0.55, 2.91]	0.590	2.03 [0.99, 4.17]	0.053	2.16 [0.92, 5.09]	0.079
Temperature day 0								
< 37.5 °C^a^								
≥ 37.5 °C	1.12 [0.71, 1.78]	0.625	1.18 [0.75, 1.85]	0.473	1.11 [0.51, 2.41]	0.794	1.40 [0.69, 2.82]	0.351
Temperature day 1								
< 37.5 °C^a^								
≥ 37.5 °C	0.65 [0.40, 1.06]	0.084	0.45 [0.24, 0.85]	0.014	0.45 [0.18, 1.12]	0.087	0.21 [0.06, 0.82]	0.025
Parasitemia day 0					-			
< 50000^a^								
≥ 50,000	1.07 [0.62, 1.83]	0.808	0.96 [0.52, 1.76]	0.886	1.13 [0.45, 2.85]	0.801	1.04 [0.50, 2.17]	0.917
Parasitaemia day 3								
Yes^a^								
No	0.05 [0.01, 0.40]	0.004	0.04 [0.01, 0.38]	0.005	0.02 [0.01, 0.10]	< 0.001	0.01 [0.00, 0.03]	< 0.001
Vomit at least once								
No^a^								
Yes	0.62 [0.24, 1.61]	0.324	0.67 [0.29, 1.54]	0.346	0.48 [0.12, 1.93]	0.301	0.49 [0.14, 1.73]	0.270

^a^Reference category

### Multivariable cox regression analysis of factors associated with treatment failure

Variables included in the multivariable cox regression analysis were gender, age group, ecological zone, axillary temperature on day 0, axillary temperature on day 1, parasite density on day 0, parasitaemia on day 3, drug type, and vomiting at least once. The risk of PCR-uncorrrected treatment failure, using the overall pooled data, was significantly higher among patients less than 5 years old compared with those aged 5 years and above (HR = 1.55; 95% CI: 1.15–2.09; p = 0.004); significantly higher in the coastal zone compared with the savannah zone (HR = 1.27; 95% CI: 1.04–1.55; p = 0.021); significantly lower among patients with axillary temperature ≥ 37.5 °C on day 1 compared with those with axillary temperature < 37.5 °C (HR = 0.66; 95% CI: 0.49–0.90; p = 0.009); significantly lower among patients with no parasitaemia on day 3 compared with those with parasitaemia (HR = 0.08; 95% CI: 0.02–0.35; p < 0.001); and significantly lower among patients who received ASAQ treatment compared with those who received AL treatment (HR = 0.36; 95% CI: 0.21–0.62; p < 0.001) (Table 3).

Using the separately pooled data for ASAQ, risk of PCR-uncorrected treatment failure was significantly higher among patients less than 5 years old compared with those aged 5 years and above (HR = 1.84; 95% CI: 1.48–2.28; p < 0.001); and significantly lower among patients with no parasitaemia on day 3 compared with those with parasitaemia (HR = 0.15; 95% CI: 0.03–0.72; p = 0.017) (Table 4).

Analysis of the pooled AL data showed risk of PCR-uncorrected treatment failure to be significantly lower among patients with axillary temperature ≥ 37.5 °C on day 1 compared with those with temperature < 37.5 °C (HR = 0.45; 95% CI: 0.24–0.85; p = 0.014); and significantly lower among patients with no parasitaemia on day 3 compared with those with parasitaemia (HR = 0.04; 95% CI: 0.01–0.38; p = 0.005) (Table 5).

The risk of PCR-corrected treatment failure, using the overall pooled data, was significantly higher in the coastal zone compared with the savannah zone (HR = 1.89; 95% CI: 1.52–2.37; p < 0.001); lower among patients with no parasitaemia on day 3 compared with those with parasitaemia (HR = 0.02; 95% CI: 0.01–0.13; p < 0.001); and significantly lower among patients who received ASAQ treatment compared with those who received AL treatment (HR = 0.24; 95% CI: 0.11–0.53; p < 0.001) (Table 3). Using the separately pooled data for ASAQ, risk of PCR-corrected treatment failure was significantly higher among patients with axillary temperature ≥ 37.5 °C on day 1 compared with those with axillary temperature < 37.5 °C (HR = 3.96; 95%; CI: 1.61–9.75; p = 0.003); and significantly lower among patients with no parasitaemia on day 3 compared with those with parasitaemia (HR = 0.05; 95% CI: 0.01–0.48; p = 0.009) (Table 4). Analysis of the pooled AL data showed risk of PCR-corrected treatment failure to be significantly lower among patients with axillary temperature ≥ 37.5 °C on day 1 compared with those with temperature < 37.5 °C (HR = 0.21; 95% CI: 0.06–0.82; p = 0.025); and significantly lower among those with no parasitaemia on day 3 compared with those with parasitaemia (HR = 0.01; 95% CI: 0.00–0.03; p < 0.001) (Table 5).

## Discussions

Routine clinical surveillance on the therapeutic efficacy of artemisinin-based combination therapy in Ghana has continued since their introduction in 2005 with support from the Global Fund. There have also been a couple of studies with support from the U.S National Institutes of Health and the U.S Navy. This paper is the first to pool all ASAQ and AL therapeutic efficacy surveillance data collected between 2005 and 2018 to look at trends and predictive factors for treatment failure among children with uncomplicated malaria in Ghana.

The study has shown that day 3 parasitaemia, which is an indicator of artemisinin or partial resistance [22] has remained less than 1% with no statistically significant increase in proportions between 2005 (0.2%) and 2018 (0.6%). This finding suggests that artemisinin remains a viable component of combination therapy in the management of uncomplicated malaria in Ghana, and compares well with findings in the sub-region [23–25].

PCR-corrected treatment failures over the years have remained less than 5% for both ASAQ and AL suggesting that Ghana, as other countries in the sub-region, has not reached the failure threshold of 10% necessary for treatment policy change (Additional file 2) [22, 23, 25–30]. This is against a backdrop of use of only one molecular marker (*msp2*) to distinguish between reinfection and recrudescence during the surveillance period between 2014 and 2018 [13, 20]. It has been suggested that using fewer markers to distinguish reinfection from recrudescence could lead to underestimation of efficacy because classification of recrudescence requires that at least one allele on every locus is common in parasites on day of enrolment (day 0) and day of parasite recurrence [31]. The risk of treatment failure among children receiving AL was 64% and 76% higher, in terms of PCR-uncorrected and PCR-corrected failures, respectively, compared with children receiving ASAQ. These findings compare well with other studies that have shown that parasite recurrence and recrudescence are commoner among patients receiving AL compared with those receiving ASAQ [26, 32].

Risk of PCR-corrected treatment failure was generally 89% higher in the coastal zone compared with the savannah zone. This finding suggests that the coastal zone represented by two cities (Cape-Coast and Accra) appears to be a hotspot for the spread of drug resistant parasites. The relatively high cosmopolitan nature of the sites within the coastal zone is likely to have resulted in a higher level of human population movement which has the potential of facilitating genetic recombination and subsequent phenotypic traits of reduced drug susceptibility within the zone [33–37].

Generally, risk of PCR-uncorrected and PCR-corrected treatment failures were respectively, 92 and 98% higher among children with parasitaemia on day 3 compared with those without parasitaemia. The high risk of treatment failure, either PCR-uncorrected or PCR-corrected, associated with parasitaemia on day 3 was observed after treatment with both ASAQ and AL, suggesting that parasitaemia on day 3 is a key predictor of either PCR-uncorrected or PCR-corrected treatment failure following ACT treatment.

Risk of treatment failure following treatment with ASAQ was about four times higher among children with axillary temperature ≥ 37.5 °C on day 1 post-treatment compared with those with temperature < 37.5 °C. On the contrary, risk of failure following treatment with AL was 79% greater among children with temperature < 37.5 °C on day 1 post-treatment compared with those with temperature ≥ 37.5 °C. Amodiaquine is a 4-aminoquinoline derivative with anti-inflammatory properties in addition to antimalarial properties compared with Lumefantrine, which is a fluorine with mainly antimalarial properties [38, 39]. It is therefore expected that ASAQ will achieve a better effect on body temperature in the presence of optimal anti-malarial activity, and so high axillary temperature (≥ 37.5 °C) on day 1 post-treatment should give an indication of possible failure during the 28-day period following treatment with ASAQ.

## Conclusions

Failure rates following treatment of Ghanaian children with uncomplicated malaria using ASAQ and AL have remained less than 5% between 2005 and 2018 warranting their continuous use in the country. Children at higher risk of treatment failure have been those receiving AL; those with parasites on day 3; those residing within the coastal zone; those with axillary temperature ≥ 37.5 °C on day 1 post-treatment (for ASAQ); and those with axillary temperature < 37.5 °C on day 1 (for AL). These predictors of treatment failure should guide management of uncomplicated malaria in Ghana.

## Supplementary Information

**Additional file 1. **Proportional hazard assumption test results.

**Additional file 2.** Kaplan Meier survival curve for PCR-corrected ASAQ and AL treatment failure.

## Data Availability

Data supporting the conclusions presented have been included in the article. The dataset analysed will be made available upon reasonable request to the corresponding author.

## Abbreviations

ACT: Artemisinin-based combination therapy
AL: Artemether-lumefantrine
ASAQ: Artesunate-amodiaquine
CPHM: Cox proportional hazard model
DHAP: Dihydroartemisinin-piperaquine
DOT: Directly observed therapy
ETF: Early treatment failure
GF: Global fund
HBHI: High burden high impact
HR: Hazard ratio
KM: Kaplan Meier
LCF: Late clinical failure
LPF: Late parasitological failure
NAMRU-3: Naval Medical Research Unit-3
NIH: National Institutes of Health
NMCP: National Malaria Control Programme
PCR: Polymerase chain reaction
RBM: Roll back malaria
SSA: Sub-saharan Africa
UG-NMIMR: University of Ghana Noguchi Memorial Institute for Medical Research
UNICEF: United Nations Children’s Fund
WHO: World Health Organization
