# Supplementary material for: Trends and predictive factors for treatment failure following artemisinin-based combination therapy among children with uncomplicated malaria in Ghana: 2005–2018
Source: BMC Infect Dis. 2021 Dec 15;21:1255. doi: 10.1186/s12879-021-06961-4 (PMC8672499; doi:10.1186/s12879-021-06961-4)
Supplement: Supplementary file 1 — Additional file 1. Proportional hazard assumption test results. [file 12879_2021_6961_MOESM1_ESM.docx]

Additional file 1 Proportional hazard assumption test results

| **Characteristics** | **Chi-square** | **df** | **p-value** |
| --- | --- | --- | --- |
| Gender |  |  |  |
| Female |  |  |  |
| Male | 2.416 | 1 | 0.120 |
| Age group (yrs) |  |  |  |
| ≥ 5 |  |  |  |
| < 5 | 2.908 | 1 | 0.088 |
| Ecological zone |  |  |  |
| Savannah |  |  |  |
| Forest |  |  |  |
| Coastal | 3.580 | 2 | 0.167 |
| Drug Type |  |  |  |
| AL |  |  |  |
| ASAQ | 2.920 | 1 | 0.088 |
| Temperature (Day 0) |  |  |  |
| < 37.5^o^C |  |  |  |
| ≥ 37.5^o^C | 0.303 | 1 | 0.582 |
| Temperature (Day 1) |  |  |  |
| < 37.5^o^C |  |  |  |
| ≥ 37.5^o^C | 0.914 | 1 | 0.339 |
| Vomit at least once |  |  |  |
| No |  |  |  |
| Yes | 1.282 | 1 | 0.258 |
| Parasitaemia Day 0 |  |  |  |
| < 50,000 |  |  |  |
| ≥ 50,000 | 2.750 | 1 | 0.097 |
| Day 3 parasitaemia |  |  |  |
| Yes |  |  |  |
| No | 3.077 | 1 | 0.079 |
|  |  |  |  |
| Overall | 15.895 | 10 | 0.103 |
