# Supplementary figures and images for: Trends and predictive factors for treatment failure following artemisinin-based combination therapy among children with uncomplicated malaria in Ghana: 2005–2018
Source: BMC Infect Dis. 2021 Dec 15;21:1255. doi: 10.1186/s12879-021-06961-4 (PMC8672499; doi:10.1186/s12879-021-06961-4)

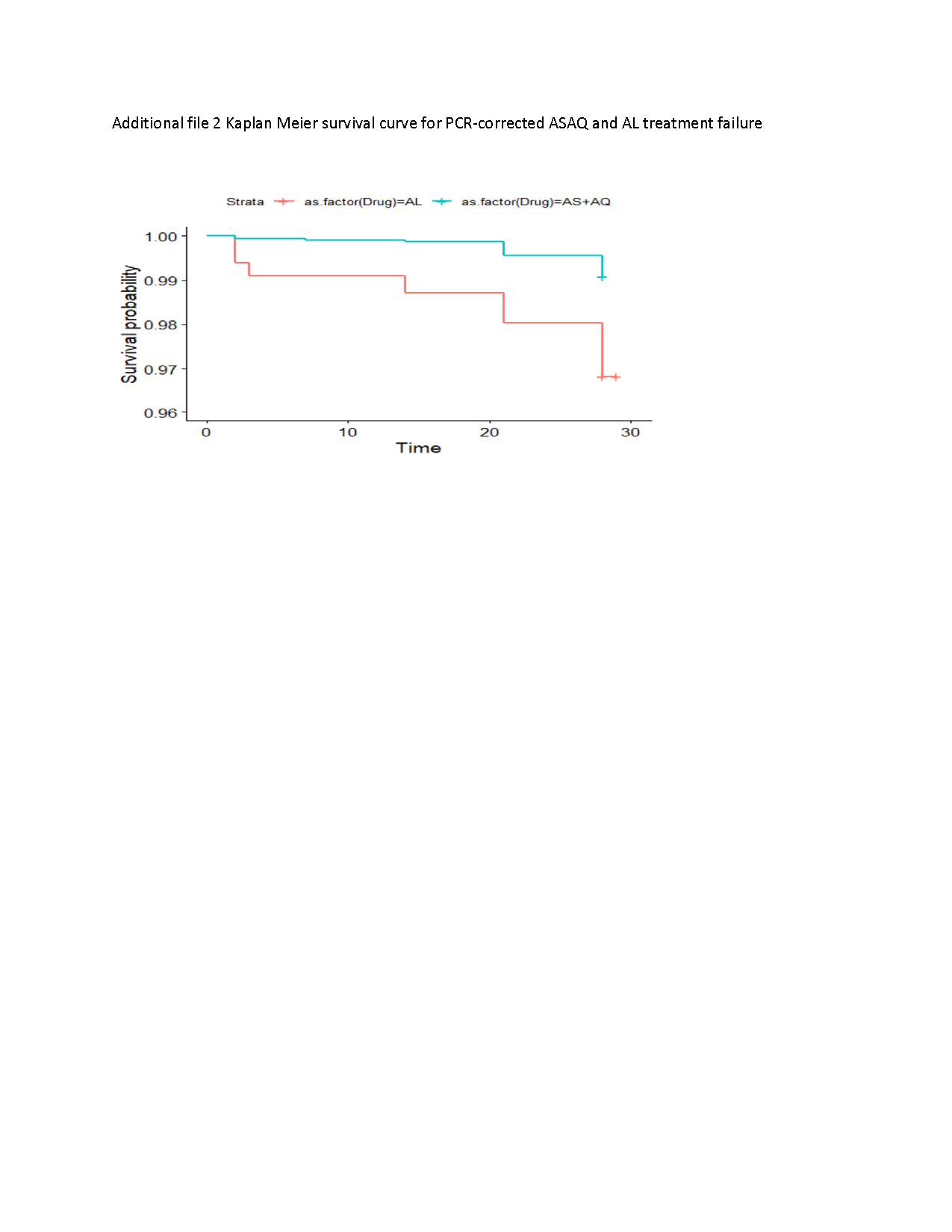

Supplement: Supplementary file 2 — Additional file 2. Kaplan Meier survival curve for PCR-corrected ASAQ and AL treatment failure. [file 12879_2021_6961_MOESM2_ESM.tiff]
